# Higher prevalence of psoriatic arthritis in the adult population in Spain? A population-based cross-sectional study

**DOI:** 10.1371/journal.pone.0234556

**Published:** 2020-06-17

**Authors:** Antonio Romero Pérez, Rubén Queiro, Daniel Seoane-Mato, Eduard Graell, Eugenio Chamizo, Lara Chaves Chaparro, Sara Rojas Herrera, Jordi Pons Dolset, Miguel A. Polo Ostáriz, Susana Ruiz-Alejos Garrido, Cristina Macía-Villa, Ana Cruz-Valenciano, María L. González Gómez, Carlos Sánchez-Piedra, Federico Díaz-González, Sagrario Bustabad-Reyes, on behalf of the Proyecto EPISER2016 Working Group

**Affiliations:** 1 Department of Rheumatology, Complejo Hospitalario de Jaén, Jaén, Spain; 2 Department of Rheumatology, Hospital Universitario Central de Asturias, Oviedo, Asturias, Spain; 3 Research Unit, Spanish Society of Rheumatology, Madrid, Spain; 4 Department of Rheumatology, Hospital Universitari Parc Taulí, Sabadell, Barcelona, Spain; 5 Department of Rheumatology, Hospital de Mérida, Mérida, Badajoz, Spain; 6 Department of Rheumatology, Fundación Hospital Calahorra, Calahorra, La Rioja, Spain; 7 Department of Rheumatology, Hospital Universitario Severo Ochoa, Leganés, Madrid, Spain; 8 Department of Rheumatology, Hospital El Escorial, San Lorenzo de El Escorial, Madrid, Spain; 9 Universidad de La Laguna, La Laguna, Santa Cruz de Tenerife, Spain; 10 Department of Rheumatology, Hospital Universitario de Canarias, La Laguna, Santa Cruz de Tenerife, Spain; Faculty of Science, Ain Shams University (ASU), EGYPT

## Abstract

**Objective:**

The prevalence of psoriatic arthritis (PsA) is very heterogeneous. There are no data on its frequency in the general population in Spain. The aim of EPISER2016 study was to estimate the prevalence of PsA in people aged ≥20 years in Spain.

**Methods:**

Cross-sectional multicenter population-based study. Subjects from all the autonomous communities in Spain were randomly selected using multistage stratified cluster sampling. Participants in each of the municipalities randomly selected for the study were administered a telephone-based questionnaire to screen for the study diseases. If the participant reported being previously diagnosed, rheumatologists from the participant’s reference hospital confirmed the diagnosis based on a review of the clinical history. Subjects not previously diagnosed but whose screening result was positive based on symptoms received a second telephone call from the investigating rheumatologist in order to evaluate the suspicion. If the suspicion remained, an appointment was made at the reference hospital to complete the diagnostic confirmation process according to CASPAR criteria. To calculate the prevalence and its 95% confidence interval (CI), the sample design was taken into account and weighing was calculated considering age, sex and geographic origin.

**Results:**

The sample comprised 4916 subjects. The prevalence of PsA was 0.58% (95%CI: 0.38–0.87). All but 1 of the 27 cases (96.30%) had been diagnosed prior to EPISER2016.

**Conclusion:**

The prevalence of PsA in Spain was among the highest reported to date, only below that reported in Norway (0.67%) and slightly higher than that reported in Italy (0.42%).

## Introduction

Psoriatic arthritis (PsA) is a chronic inflammatory joint disease associated with psoriasis. PsA can lead to structural joint damage, which is in turn related to disability and high direct and indirect costs, with major implications for health policy [1]. PsA is associated with poorer health-related quality of life, which is characterized by physical pain and functional limitations in activities of daily living [2].

Given the burden that a disease such as PsA generates for the health system [1], it is important to measure its frequency in the general population in order to improve planning of care for these patients. The prevalence is very heterogeneous, with figures ranging from 0.02% in Sweden to 0.67% in Norway. A recent meta-analysis analyzed the factors underlying this disparity and found that the significant factors (in order of importance) were as follows: the criterion used to define PsA, the geographical area (higher prevalence in northern countries and lower in Asia and South America), and the data collection period (estimated prevalence is lower in studies carried out before 1999) [3].

We haven´t found epidemiological studies analyzing the prevalence of PsA in Spain. Population-level data are available for the prevalence of psoriasis in Spain (2.3%) [4] and for the prevalence of PsA in patients with psoriasis (22.9%) [5]. These data would not be sufficiently valid to enable an appropriate estimation of the prevalence of PsA in the general population. On the one hand, patients with arthritis who did not have psoriasis would be excluded, even if they had a family history of psoriasis; on the other, these data would not include patients with psoriasis who did not come into contact with the health system. Therefore, they probably underestimate the real prevalence of PsA. The main objective of the EPISER2016 study, which was conducted by the Spanish Society of Rheumatology, included the estimation of the prevalence of PsA in general population aged ≥20 years in Spain.

## Materials and methods

The methods and characteristics of the sample of EPISER2016 have been described elsewhere [6,7]. Briefly, the study was a population-based multicenter cross-sectional study that aimed to estimate the prevalence of rheumatic diseases (rheumatoid arthritis; SLE; symptomatic osteoartrhritis of the hand, knee, hip, cervical and lumbar spine; fibromyalgia; ankylosing spondylitis; psoriatic arthritis; Sjögren’s syndrome; gout; and symptomatic osteoporotic fracture) in adult population in Spain. Assuming a Poisson distribution, a sample comprising 4,000 individuals would enable to obtain a 95% confidence interval (CI) of 0.30–0.77 for a prevalence of 0.5% (expected for rheumatoid arthritis) and of 0.14–0.54 for a prevalence of 0.3% (expected for psoriatic arthritis). Assuming 20% of missing values, it was considered necessary to include around 5,000 individuals.

Subjects were randomly selected by means of multistage stratified (strata based on rural/urban municipalities, sex and age in accordance with the distribution of the population in Spain) cluster sampling, who resided in 78 municipalities randomly selected throughout all the autonomous communities in Spain.

From November 2016 to October 2017, the participants in each municipality were contacted using random digit dialing and a Computer Assisted Telephone Interviewing system (CATI) to conduct a questionnaire for the screening of the diseases under study. The survey was mostly performed via landlines, but in order to facilitate access to younger population and expand the registry, we incorporated mobile phones since March 2017, which represent 20.3% of the final sample. This figure reflects the proportion of homes in Spain that relied solely on a mobile telephone connection. An external sociological research company with experience in the field of health care and with call center service (Ipsos España) implemented both the random selection of telephone numbers in each municipality and the initial screening interviews. In case of non-answered phone calls, a minimum of 6 attempts were made in different time frames. If after these attempts there was no answer or the subject refused to participate, another phone number within the same municipality was randomly selected [7].

Screening was based on 2 complementary paths for all the participants, namely, the subject was asked if he/she had already been diagnosed with PsA, and symptom-based screening was also performed (S1 File).

**Fig 1:**
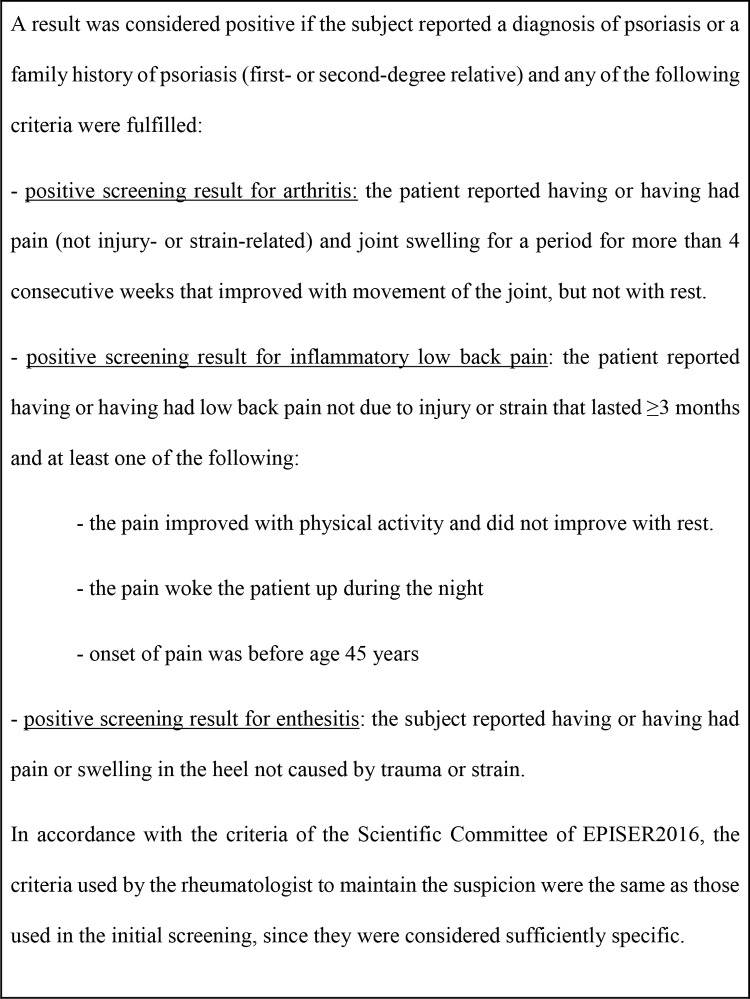
Definition of positive symptom-based screening for psoriatic arthritis.

If a participant reported having been diagnosed, his/her consent was requested so that the investigating rheumatologists from the reference hospital of the municipality could confirm the presence of the diagnosis in his/her clinical history. Participants not previously diagnosed but who had a positive result in symptom-based screening (Fig 1) received a second telephone call from the investigating rheumatologist to evaluate the suspicion by means of a second questionnaire (S1 File). Those participants for whom the suspicion remained after the second telephone call were given an appointment at their reference hospital to complete the diagnostic confirmation process (physical examination and additional tests), according to the CASPAR criteria [8]. These were applied to confirm those cases not diagnosed before the study. In the case of previously diagnosed cases, there was no request to actively verify that they fulfilled the criteria according to their clinical history; clearly identified diagnoses were accepted irrespective of the criteria applied.

Subjects who completed the call center interview with a positive result for the screening of PsA and the rheumatologist could not access their clinical records and/or contact them to confirm or rule out the diagnosis were considered missing.

Oral informed consent was required from all participants during the first telephone call. Written informed consent was also requested from all those participants who came to the participating centers to undergo the physical examination and additional tests. Approval was obtained from the Research Ethics Committee (REC) of Hospital Universitario de Canarias (approval number: Acta 12/2016), which acted as the reference REC, and from the RECs of those participating centers that required to approve the study locally (Hospital Universitario Nuestra Señora de Candelaria, Hospital General Universitario de Elda, Complejo Asistencial Universitario de Palencia, Complejo Hospitalario de Navarra, Hospital Universitario Severo Ochoa, Hospital Universitario Central de Asturias, Hospital Universitario Germans Trias i Pujol, Hospital Universitari Parc Taulí). The study was performed in line with the principles of the Declaration of Helsinki. The use of oral consent was approved by the RECs. At the beginning of the phone call, the interviewer introduced the study to the individual and asked for his/her permission to answer the questionnaire. Once it was completed, the individual was informed about the next steps of the study and the use of the information. Only those who gave their consent were considered participants and part of the study sample. Their consent was recorded on audio.

### Statistical analysis

Prevalence and the 95% CI were calculated taking into account the design of the sample; weighting was applied depending on the probability of selection at each of the sampling stages, taking as a reference the distribution of the population in Spain in 2016 according to Continuous Register Statistics from the Spanish National Statistical Institute (www.ine.es). Weighting was calculated based on age (grouped by decades), sex, and geographic origin (3 areas were defined: North [Galicia + Asturias + Cantabria + Basque Country + Navarra + La Rioja], Mediterranean and Canary Islands [Catalonia + Comunidad Valenciana + Balearic Islands + Murcia + Andalusia + Canary Islands], and Center [Comunidad de Madrid + Castilla y León + Aragón + Castilla-La Mancha + Extremadura]); based on these characteristics each individual in the sample represented a certain number of individuals in the population (S3 File).

We also performed a sensitivity analysis including as PsA cases those subjects who were missing.

Due to feasibility issues, the validity of the screening questionnaire could not be evaluated prior to the study. Nevertheless, we calculated the positive predictive value (PPV) of the questionnaire taking into account the cases confirmed subsequently. In addition, we carried out a substudy of the negative predictive value (NPV) in a sample of 209 subjects randomly selected from those who had a negative screening result for all of the diseases studied in EPISER2016. These subjects were contacted by telephone by the research rheumatologist in their catchment area with the aim of confirming the negative result. If it was not possible to rule out a positive result in the phone call, the patient was attended for examination and performance of the relevant additional tests according to the disease suspected.

The analyses were performed using IBM SPSS Statistics v22.

## Results

A total of 84,098 different phone numbers were dialed. Of them, 50,170 were wrong numbers or were unanswered; 28,784 individuals refused to participate (96.9% of them refused at the very beginning of the interview), and 5144 complete interviews were made. The response rate, once the individual had been contacted, was 15.2%.

**Fig 2:**
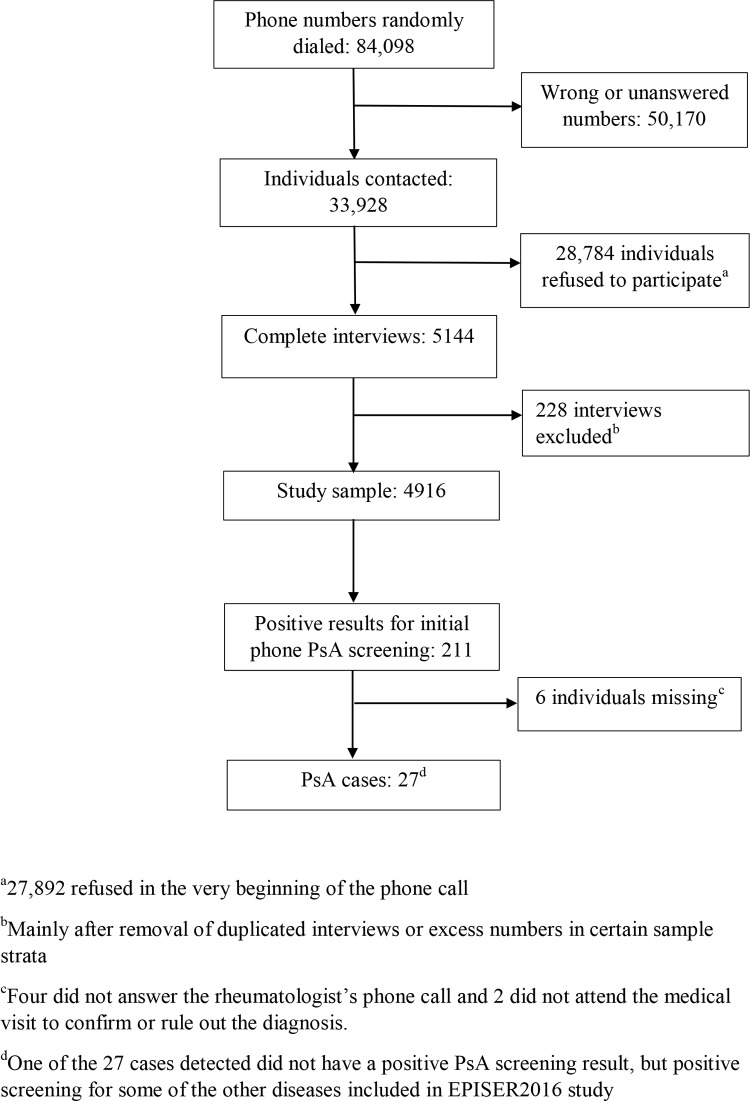
Results of the recruitment and case detection.

The final sample comprised 4916 individuals, mainly after removal of duplicated interviews or excess numbers in certain sample strata (Fig 2). Baseline characteristics of the whole sample and a comparison with the general population aged 20 years or older in Spain (reference population in EPISER2016) have been published in detail elsewhere [7]. The sample can be considered representative of the population aged 20 years or over in Spain for the aim of the study [7].

### Predictive values of PsA screening

Of the 4916 subjects interviewed, 211 (4.3%) were positive for PsA screening after the interview from the call center. Of these, 6 (2.84%) were missing. In 20 of the 28 participants who reported being previously diagnosed, the rheumatologist confirmed the diagnosis in the review of the clinical history (positive predictive value [PPV], 71.4%).

Of the 184 participants with a positive symptom-based screening result and who had not been diagnosed before EPISER2016, 4 did not answer the rheumatologist’s phone call. Furthermore, in 10 cases, the rheumatologist continued to suspect psoriasis after the telephone interview and, therefore, made an appointment. Two of the 10 subjects did not attend the visit, and the diagnosis of PsA was confirmed in 1 of the remaining 8 participants.

The PPV of the screening for PsA carried out by the call center was 12.68% (26 cases among 205 subjects with a positive screening result who completed the study). One of the 27 cases detected did not have a positive PsA screening result: in the call center interview, the subject reported being previously diagnosed with rheumatoid arthritis and, even though he/she reported being diagnosed with psoriasis, symptom-based screening was positive for fibromyalgia and for osteoarthritis of the hands. Based on the review of the clinical history, it was subsequently confirmed that the subject was diagnosed with PsA prior to the study.

In the pre-planned substudy on 209 subjects randomly selected among those with a negative screening result for all RMDs studied in EPISER2016, negative predictive value of the whole questionnaire for PsA was 100%.

### Prevalence of PsA

The estimated prevalence of PsA was 0.58% (95% CI: 0.38–0.87).

All but 1 of the 27 cases (96.30%) had been diagnosed prior to EPISER2016. 81.5% (22/27) fulfilled the CASPAR criteria. Of the 5 remaining cases, the investigating rheumatologists were unable to confirm that the criteria were fulfilled in 3 cases, although they did confirm that the patients had been diagnosed in their department before the study. For the other 2 cases, the rheumatologists confirmed that the patients had PsA but did not fulfill the CASPAR criteria. One of these 2 patients was diagnosed as a result of the study.

In the sensitivity analysis, considering the 6 missing subjects as PsA cases, prevalence was 0.7%, which falls within the 95% CI.

### Characteristics of the PsA cases

The most important sociodemographic characteristics are shown in Table 1. Of the 27 cases, 13 (48.1%) were women. 20 (74.1%) had overweight or obesity.

**Table 1:** Association between presence of psoriatic arthritis and sociodemographic, anthropometric and lifestyle variables. Bivariate analysis.

Variable	PsA cases (n = 27)	Subjects without PsA (n = 4883)	p-value^a^
Age			0.061
• 20–39	11.1%	32.3%	
• 40–59	48.1%	38.4%	
• 60-	40.7%	29.4%	
Sex, female	48.1%	54.5%	0.512
Area of Spain			0.658
• North	33.3%	28.7%	
• Mediterranean and Canary Islands	33.3%	42.0%	
• Center	33.3%	29.3%	
Educational level			0.045
• Basic	44.4%	37.2%	
• Intermediate	40.7%	25.9%	
• Higher	14.8%	36.9%	
Body mass index			0.218
• Normal weight (18.5 ≤BMI <25)	25.9%	44.6%	
• Underweight	0%	1.1%	
• Overweight (25 ≤ BMI < 30)	51.9%	39.5%	
• Obesity (BMI ≥30)	22.2%	14.8%	
Smoking habit			0.450
• Never smoker	37.0%	49.2%	
• Former smoker	33.4%	26.7%	
• Smoker	29.6%	24.1%	
Born abroad	7.4%	7.0%	0.713
Residence in an urban municipality	85.2%	77.4%	0.335

^a^Bivariate logistic regression analysis.

## Discussion

The prevalence of PsA in the general adult population is high (0.58%) in Spain. It is only lower than the prevalence reported in Norway (0.67%) and Lithuania (0.64%) [9,10] and slightly higher than that reported in another southern European country, Italy (0.42%) [11]. These studies are comparable in terms of their design (sample survey, use of telephone or postal screening, and well-defined diagnostic criteria). In the case of the study performed in Lithuania, the overall prevalence reported was for spondyloarthritis. Even taking into account the lower limit of our confidence interval (0.38%), our results are still higher than those reported in most studies published elsewhere [3].

The use of different criteria to define a case is a significant cause of the disparate results reported in the various prevalence studies [3]. On the one hand, several standardized and validated classification criteria are available; on the other, criteria agreed by the investigators to facilitate the estimation of prevalence are also used (eg, ICD codes, self-reported diagnosis, presence of arthritis + psoriasis). Some criteria do not admit a diagnosis of PsA with positive rheumatoid factor, whereas others require the presence of psoriasis and do not include enthesitis or diseases associated with psoriasis. Lastly, specific nonclassifiable subgroups of PsA (SAPHO syndrome, psoriatic onychopachydermoperiostitis, mutilating forms, paucisymptomatic axial forms) may go undetected, thus reducing the estimated prevalence.

Of the various criteria validated for PsA, the CASPAR criteria are the currently recommended option, both for research and for clinical practice. Their internal validity has been confirmed in early diagnosed and well-evolved cases, thus making them the criteria of choice in the most recent studies [10,12,13–16]. These criteria were used in EPISER2016, although other criteria in the case of previous diagnoses, such as diagnosis made by the attending rheumatologist, were permitted, thus making room for rarer forms of PsA. This strategy is similar to that employed in the study by Hoff et al. [10] in Norway, where 95.6% (323/338) of the cases fulfilled the CASPAR criteria.

Some prevalence studies select regions that—because of their characteristics—are defined as being representative of the whole population. This choice facilitates the study and may prove advantageous in terms of improving coordination between hospitals and training of rheumatologists/surveyors, thus reducing a variability factor. However, the variability in PsA by geographic region can be significant. In Norway, results differ between the center and the east of the country when the studies of Hoff et al. [10] and Madland [17] are compared, with a prevalence of 0.67% and 0.19%, respectively. Therefore, the estimated prevalence of PsA in a population should be approached with caution when this is reported for very local areas. The fact that the sample in EPISER2016 was taken from all of the regions of Spain would have minimized area-dependent variability. The only studies on the prevalence of PsA to sample by area were those of Gelfand et al. [18] in the USA and Ogdie et al. [19] in the UK, although the data sources were from self-reported diagnoses in the former and dispensed drug registries in the latter.

Another factor to consider when comparing prevalence figures of PsA between studies is the data collection period [3]. In this sense, studies from different parts of the world have observed a sustained increase in recent years [20,21]. This fact could contribute to explain some of the higher figure estimated in EPISER2016 with respect to previous studies.

Performance of a population-based study on the prevalence of PsA is an epidemiological challenge that is subject to a series of difficulties: the inherent clinical heterogeneity and polymorphisms, the effect of genetics and geographic area on the disease, and hamper diagnosis. In order to overcome these difficulties, EPISER2016 has envisaged the following strategies: stratified sampling that included different geographic areas; randomized 2-stage screening surveys, with the participation of a rheumatologist; use of highly sensitive and specific validated criteria for defining a case (currently the CASPAR criteria); and inclusion of the possibility of diagnosis made by the attending rheumatologist.

Lastly, the response rate for calls in EPISER2016 was 15.2%, which could be interpreted as a possible source of bias. In the last decades epidemiological studies in developed countries have been hampered by a marked decline in participation levels, and thus their importance in terms of the validity of estimates remains an open question. Different reports about this issue have concluded that low response rates do not necessarily induce a non-response bias on their own [22–26]. The response rate in EPISER2016 is consistent with the most recent estimations for telephone surveys and could have decreased compared to other studies due to the demanding sampling requirements (strata based on rural/urban, sex and decades of age) [26–28]. These requirements, together with the similarity in the characteristics analyzed between the sample and the general population aged ≥20 years in Spain (reference population in EPISER2016), and the weighting used in the estimation of the prevalence figures, would indicate that the low response rate did not lead to a significant bias that invalidated the estimated prevalence [7,26].

During the design of EPISER2016 study other alternatives were considered, but phone calls were the best alternative in our context for access to the general population. In Spain there are no administrative claims that allow to estimate the prevalence for the whole country. There could be other alternatives to phone calls for recruitment, such as postal mail, but the logistics were more complicated and a better response rate was not assured beforehand.

In conclusion, the estimated prevalence of PsA in the general adult population in Spain is 0.58% (95% CI 0.38–0.87). This prevalence is among the highest reported to date, only below that reported in Norway and slightly higher than that reported in Italy. Most of the cases detected in EPISER2016 were classified based on the CASPAR criteria, which are currently the most widely used and recommended. Given the burden that a disease such as PsA generates for the health system, it is important to measure its frequency in the general population in order to improve planning of care for these patients.

### Non-author members of EPISER2016 study group

Lucía Silva-Fernández, Francisco J. Blanco (Complejo Hospitalario Universitario de A Coruña), Antonio Juan-Mas (Hospital Son LLàtzer), José M. Pego-Reigosa (Complejo Hospitalario Universitario de Vigo), Javier Narváez (Hospital Universitario de Bellvitge), Francisca Sivera, Neus Quilis Martí (Hospital General Universitario de Elda), Raúl Cortés Verdú (Hospital General de Ontinyent), Fred Antón-Pagés (Complejo Asistencial Universitario de Palencia), Víctor Quevedo Vila (Hospital Comarcal de Monforte de Lemos), Laura Garrido Courel, Natividad del Val del Amo, Inmaculada Paniagua Zudaire (Complejo Hospitalario de Navarra), Gustavo Añez Sturchio, Fermín Medina Varo, María del Mar Ruiz Tudela (Hospital Universitario Puerta del Mar), Javier Ballina, Anahy Brandy García (Hospital Universitario Central de Asturias), Dolores Fábregas Canales (Hospital de Barbastro), Teresa Font Gayá, Carolina Bordoy Ferrer (Hospital Comarcal de Inca), Beatriz González Álvarez, Laura Casas Hernández, Fátima Álvarez Reyes, Mónica Delgado Sánchez (Hospital Universitario Nuestra Señora de la Candelaria), Cristina Martínez Dubois (Hospital Universitario Marqués de Valdecilla), Simón Ángel Sánchez-Fernández, Luisa Marena Rojas Vargas, Paula Virginia García Morales (Complejo Hospitalario Mancha Centro), Alejandro Olivé, Paula Rubio Muñoz (Hospital Universitari Germans Trias i Pujol), Marta Larrosa, Noemí Navarro Rico (Hospital Universitari Parc Taulí), Mercedes Morcillo Valle (Hospital El Escorial), Deseada Palma Sánchez, María José Moreno Martínez, Marta Mayor González (Hospital General Universitario Rafael Méndez), Fernando Pérez Ruiz, Joana Atxotegi Sáenz de Buruaga, Irati Urionagüena Onaindia, Boris Anthony Blanco Cáceres (Hospital Universitario Cruces). Lead author: Sagrario Bustabad-Reyes (e-mail: sagrario.bustabad@gmail.com).

## Supporting information

S1 File Telephone screening questionnaires. (PDF)

S2 File Data set. (XLSX)

S3 File Syntax used for weighting and prevalence estimation. (PDF)
