# Supplementary material for: Higher prevalence of psoriatic arthritis in the adult population in Spain? A population-based cross-sectional study
Source: PLoS One. 2020 Jun 17;15(6):e0234556. doi: 10.1371/journal.pone.0234556 (PMC7299392; doi:10.1371/journal.pone.0234556)
Supplement: S1 File — (PDF) [file pone.0234556.s001.pdf]

SUPPLEMENTARY MATERIAL

CALL CENTER QUESTIONNAIRE TO SCREEN FOR PsA SYMPTOMS

ARTHRITIS SCREENING

1. Do you have or have you had painful joints that were not caused by a fall/injury or strain?
1. Yes 0. No

If the answer to the first question is "yes", continue with:

2. For more than 4 weeks in a row? 1. Yes 0. No

If the answer to the second question is "yes", continue with:

3. Has the joint(s) ever felt inflamed or swollen and painful for more than 4 weeks in a row?
1. Yes 0. No

4. Which of the following joints was painful for more than 4 weeks in a row (not caused by a fall/injury or strain)?

a. Hands ☐ Yes ☐ No

b. Side ☐ Right ☐ Left ☐ Both

c. Hip/groin ☐ Yes ☐ No

d. Side ☐ Right ☐ Left ☐ Both

e. Knee ☐ Yes ☐ No

f. Side ☐ Right ☐ Left ☐ Both

g. Ankle ☐ Yes ☐ No

h. Side ☐ Right ☐ Left ☐ Both

i. Feet ☐ Yes ☐ No

j. Side ☐ Right ☐ Left ☐ Both

5. Hip/groin pain:
a. Does the pain get worse with exertion, walking, going up/down stairs? 1. Yes 0. No
b. Does the pain improve with physical activity/exercise *(it feels painful, but the pain is relieved by a short period of movement)*? 1. Yes 0. No
c. Does the pain improve with rest? 1. Yes 0. No

6. Knee pain:

a. Does the pain get worse with exertion, walking, or going up/down stairs? 1. Yes 0. No
b. Does the pain improve with physical activity/exercise *(it feels painful, but the pain is relieved by a short period of movement)*? 1. Yes 0. No
c. Does the pain improve with rest *(feels better when sitting or lying down)*? 1. Yes 0. No

7. Ankle pain:
a. Does the pain get worse with exertion, walking, or going up/down stairs? 1. Yes 0. No
b. Does the pain improve with physical activity/exercise (*it feels painful, but the pain is relieved by a short period of movement)*? 1. Yes 0. No
c. Does the pain improve with rest *(feels better when sitting or lying down)*? 1. Yes 0. No

8. Foot pain:
a. Does the pain get worse with exertion, walking, or going up/down stairs? 1. Yes 0. No
b. Does the pain improve with physical activity/exercise *(it feels painful, but the pain is relieved by a short period of movement)*? 1. Yes 0. No
c. Does the pain improve with rest *(feels better when sitting or lying down)*? 1. Yes 0. No

9. Hand pain:
a. Does the pain get worse when you use your hands/move your fingers? 1. Yes 0. No
b. Does the pain improve when you use your hands/move your fingers *(the pain improves after moving the hands for a short period)*? 1. Yes 0. No
c. Does the pain improve with rest *(the pain improves when the hands are resting, without exertion)*? 1. Yes 0. No

10. In the morning, are you unable to move the affected area or does it feel stiff? *(That is, when you get up in the morning, you feel stiff and fins it difficult to move your joints. Walking is difficult in the case of hip, knee, or foot pain. In the case of hand pain, it is difficult to close the hands)* 1. Yes 0. No

If the response to question 10 is "Yes", continue with:
11. For more than half an hour? 1. Yes 0. No

SCREENING FOR INFLAMMATORY LOW BACK PAIN
1. Do you feel or have you ever felt pain in the lower part of the back that was not caused by a fall/injury or strain? 1. Yes 0. No

If the answer to question 1 is "Yes", continue with:

2. Has this pain lasted 3 or more months in a row, even if it is not continuous/comes and goes? 1. Yes 0. No

If the response to question 2 is "Yes", ask:

3. Does the pain get worse if you lift something heavy or exert yourself? 1. Yes 0. No

4. Does the pain improve with physical activity/exercise *(it feels painful, but the pain is relieved by a short period of movement)*? 1. Yes 0. No

5. Does the pain improve with rest *(feels better when sitting or lying down)*? 1. Yes 0. No

6. Does the pain wake you at night? 1. Yes 0. No

7. Did this pain in the lower part of the back first appear before you were 45? 1. Yes 0. No

SCREENING FOR ENTHESITIS

1. Do you feel or have you felt pain in your heel or has it been swollen (not caused by a fall/injury or strain)? 1. Yes 0. No

Note for the interviewer: if the person mentions that he/she was told it was due to a spur, tick "No". If he/she says "sometimes" or "I think so", do not insist and tick "Yes".

HISTORY OF PSORIASIS

1. Has your doctor told you that you have or have had psoriasis? 1. Yes 0. No
2. Has a close family member (grandparents, parents, siblings, or children) been diagnosed with psoriasis? 1. Yes 0. No 9. Don't know/No answer

TELEPHONE QUESTIONNAIRE FOR ASSESSMENT OF PsA SUSPICION BY THE RHEUMATOLOGIST

The questions varied according to whether the initial screening had been positive for inflammatory low back pain, arthritis, or enthesitis. If the screening had been positive for more than 1 condition, maintaining the suspicion for 1 was sufficient.

INITIAL SCREENING POSITIVE FOR INFLAMMATORY LOW BACK PAIN

1. According to the questionnaire administered on (date of the interview from the call center), you have or have had pain in the lower part of your back that was not caused by a fall/injury or strain. Is this the case? ☐ Yes ☐ No

If the answer to question 1 is "Yes", continue with:

2. Have you felt the pain for 3 or more consecutive months, even if the pain was not continuous? ☐ Yes ☐ No

If the answer to question 2 is "Yes", continue with:

3. Does the pain improve with physical activity/exercise? ☐ Yes ☐ No

4. Does the pain improve with rest? ☐ Yes ☐ No

5. Does the pain wake you during the night? ☐ Yes ☐ No

6. Did the pain first appear before age 45? ☐ Yes ☐ No

7. Has your doctor ever told you that you have or have had:

☐ Psoriasis

☐ Inflammatory bowel disease (ulcerative colitis/Crohn disease)

☐ Uveitis (clarify if necessary: redness and pain in your eye for which you had to see an ophthalmologist and which was not caused by an infection)

☐ None of the above

8. Has a close family member (grandparents, parents, siblings, or children) ever been diagnosed with any of the following:

☐ Psoriasis

☐ Ankylosing spondylitis

☐ Inflammatory bowel disease/ulcerative colitis/Crohn disease

☐ None of the above

Taking into account the previous answers:

9. Result of screening for psoriatic arthritis[1] ☐ Positive ☐ Negative

10. Do you still suspect psoriatic arthritis? ☐ Yes ☐ No

11. Reason for not suspecting psoriasis (state whether you no longer suspect psoriatic arthritis in the case of a positive screening result according to the criteria defined by the scientific committee):

If you NO LONGER SUSPECT psoriatic arthritis:

12. Do you suspect inflammatory low back pain?

INITIAL SCREENING POSITIVE FOR ARTHRITIS

1. According to the questionnaire administered on (*date of the call center interview*), you felt pain in your (*location in the call center interview*) that was not caused by a fall/injury or strain. Is this the case? ☐ Yes ☐ No

If the answer to question 1 is "Yes", continue with:

2. Did it last for more than 4 weeks in a row? ☐ Yes ☐ No

If the answer to question 2 is "Yes", continue with:

3. Location:

Hand ☐ Yes ☐ No

Side ☐ Both ☐ Right ☐ Left

Hip ☐ Yes ☐ No

Side ☐ Both ☐ Right ☐ Left

Knee ☐ Yes ☐ No

Side ☐ Both ☐ Right ☐ Left

Ankle ☐ Yes ☐ No

Side ☐ Both ☐ Right ☐ Left

Foot ☐ Yes ☐ No

Side ☐ Both ☐ Right ☐ Left

4. Has your (*area affected*) ever felt inflamed/swollen for more than 4 weeks? ☐ Yes ☐ No

5. Does the pain in your hip/groin get worse with exertion, walking, going up/down stairs?

☐ Yes ☐ No

or

6. Does the pain improve with physical activity/exercise? ☐ Yes ☐ No

7. Does the pain in your hip/groin improve with rest? ☐ Yes ☐ No

8. Does the pain in your knee(s) worsen with exertion, walking, or going up/down stairs?

☐ Yes ☐ No

or

9. Does the pain improve with physical activity/exercise? ☐ Yes ☐ No

10. Does the pain in your knee(s) improve with rest? ☐ Yes ☐ No

11. Does the pain in your ankle get worse with exertion, walking, going up/down stairs? ☐ Yes ☐ No

or

12. Does the pain improve with physical activity/exercise? ☐ Yes ☐ No

13. Does the pain in your ankle improve with rest? ☐ Yes ☐ No

14. Does the pain in your foot get worse with exertion, walking, going up/down stairs? ☐ Yes ☐ No

or

15. Does the pain improve with physical activity/exercise? ☐ Yes ☐ No

16. Does the pain in your foot improve with rest? ☐ Yes ☐ No

17. Does the pain in your hand(s) worsen when you use your hands/move your fingers? ☐ Yes ☐ No

or

18. Does the pain in your hand(s) improve when you use your hands/move your fingers? ☐ Yes ☐ No

19. Does the pain in your hand(s) improve with rest? ☐ Yes ☐ No

20. In the morning, are you ever unable to move the affected area(s) or do you find it(them) stiff? ☐ Yes ☐ No

If the answer to question 20 is "Yes", continue with:

21. For more than half an hour? ☐ Yes ☐ No

Taking the previous answers into account:

22. Result for arthritis screening[1] ☐ Positive ☐ Negative

23. Do you continue to suspect arthritis? ☐ Yes ☐ No

24. Reason for not suspecting arthritis (state whether you no longer suspect psoriatic arthritis in the case of a positive screening result according to the criteria defined by the scientific committee):

If you suspect arthritis, continue with:

25. Has your doctor told you that you have or have had psoriasis? ☐ Yes ☐ No

26. Has a close family member (grandparents, parents, siblings, or children) been diagnosed with psoriasis? ☐ Yes ☐ No

Taking into account the previous answers:

27. Do you still suspect psoriatic arthritis? ☐ Yes ☐ No

INITIAL SCREENING POSITIVE FOR ENTHESITIS

1. Does your heel hurt, or has it hurt, or have you noticed it to be swollen (without having been caused by an injury or strain)? ☐ Yes ☐ No

If the answer to question 1 is "Yes", continue with:

2. Has your doctor ever told you that you have or have had psoriasis? ☐ Yes ☐ No

3. Has a close family member (grandparents, parents, siblings, or children) ever been diagnosed with psoriasis? ☐ Yes ☐ No

Taking into account the previous answers:

Do you still suspect psoriatic arthritis? ☐ Yes ☐ No

[1] In accordance with the criteria defined by the scientific committee: the criteria used by the rheumatologist to maintain the suspicion should be the same as those used in the initial screening, since they were considered to be sufficiently specific.

CUESTIONARIO DEL CENTRO DE LLAMADAS PARA EL CRIBADO SINTOMÁTICO DE APs

CRIBADO DE LUMBALGIA INFLAMATORIA

33. ¿Tiene o ha tenido dolor de la parte baja de la espalda/cintura/espalda a la altura de los riñones/lumbares *(es el dolor de lumbago o lumbalgia)* sin que haya sido por una caída / golpe o sobresfuerzo *(es decir, el dolor no está relacionado con ninguna caída, golpe o sobreesfuerzo)*? 1. Sí 0. No

SÓLO SI P33=1

34. ¿Ha durado ese dolor 3 o más meses seguidos, aunque el dolor no sea fijo / tenga altibajos? 1. Sí 0. No

SÓLO SI P34=1

35. ¿Empeora al coger peso o hacer esfuerzos *(si cuando levanta peso o cuando trabaja mucho, se encuentra peor)*? 1. Sí 0. No

    ENTREV: si indica que "a veces" o "parece que sí", no insistir y marcar "sí".

SÓLO SI P34=1

36. ¿Mejora con la actividad física y el ejercicio *(tiene el dolor pero después de que está un rato moviéndose, se le alivia)*? 1. Sí 0. No

    ENTREV: si indica que "a veces" o "parece que sí", no insistir y marcar "sí".

PROG.: P.35 Y 36 EN LA MISMA PANTALLA

ENTREV.: En caso de obtener la misma respuesta a ambas cuestiones (sí a las dos o no, a las dos. Confirmad con el entrevistado/a la comprensión de la pregunta)

SÓLO SI P34=1

37. ¿Mejora con el reposo *(se encuentra mejor cuando está sentado o acostado)*?

    1. Sí 0. No

    ENTREV: si indica que "a veces" o "parece que sí", no insistir y marcar "sí".

SÓLO SI P34=1

38. ¿Le despierta el dolor por la noche *(estando durmiendo, el dolor le despierta)*?

    1. Sí 0. No

SÓLO SI P34=1

39. Este dolor de la parte baja de la espalda/cintura/espalda a la altura de los riñones/lumbares *(es el dolor de lumbago o lumbalgia)*, ¿empezó antes de los 45 años?

    1. Sí 0. No

CRIBADO DE ENTESITIS

40. ¿Le duele o le ha dolido el talón *(la zona de detrás del tobillo, el calcáneo o calcañar)*, o lo ha notado hinchado (sin que haya sido por un golpe o sobresfuerzo)? 1. Sí 0. No

    ENTREV.: si la persona comenta que le dijeron que era por un espolón, marcar "No" como respuesta. Si indica que "a veces" o "parece que sí", no insistir y marcar "sí".

CRIBADO DE ARTRITIS

41. ¿Tiene o ha tenido dolor en alguna articulación sin que haya sido por una caída / golpe o un sobresfuerzo *(es decir, el dolor no está relacionado con ninguna caída, golpe o sobreesfuerzo)*? 1. Sí 0. No

SÓLO SI P41=1

42. ¿Durante más de 4 semanas seguidas? 1. Sí 0. No

SÓLO SI P42=1

43. ¿Alguna vez ha tenido inflamación o hinchazón *(aumento de volumen o mayor tamaño)* de alguna de esas articulaciones con dolor durante más de 4 semanas seguidas?
1. Sí 0. No

SÓLO SI P42=1

44.0 Sin que haya sido por una caída/golpe o un sobresfuerzo, ¿en cuál/es de las siguientes articulaciones ha tenido ese dolor durante más de 4 semanas seguidas?

ENTREV.: Respuesta sugerida. Leer opciones de respuesta

PROGR.: La variable 44.0 no debe aparecer en el fichero de datos

PROGR.: programar las variables 44, 46, 48, 50 y 52 como respuesta múltiple

				SÓLO SI CÓDIGO=1					
		**Sí**	**No**	**Ese dolor de…. (introducir respuestas con código 1 en P44, P46, P48, P50 y P52. Una pregunta por respuesta) era en..**					
	Manos	*P44= 1*	*P44= 0*	Mano derecha	*P45= 1*	Mano izquierda	*P45= 2*	Ambas manos	*P45= 3*
	Caderas/ ingles	*P46= 1*	*P46= 0*	Cadera/ ingle derecha	*P47= 1*	Cadera/ ingle izquierda	*P47= 2*	Ambas caderas/ingles	*P47= 3*
	Rodillas	*P48= 1*	*P48= 0*	Rodilla derecha	*P49= 1*	Rodilla izquierda	*P49= 2*	Ambas rodillas	*P49= 3*
	Tobillos	*P50= 1*	*P50= 0*	Tobillo derecho	*P51= 1*	Tobillo izquierdo	*P51= 2*	Ambos tobillos	*P51= 3*
	Pies	*P52= 1*	*P52= 0*	Pie derecho	*P53= 1*	Pie izquierdo	*P53= 2*	Ambos pies	*P53= 3*

SÓLO SI P42=1 Y P46=1

54. El dolor de cadera/ingle, ¿empeora con el esfuerzo, al caminar o al subir/bajar escaleras *(si cuando levanta peso o cuando trabaja mucho, se encuentra peor)*?
1. Sí 0. No

ENTREV: si indica que "a veces" o "parece que sí", no insistir y marcar "sí".

SÓLO SI P42=1 Y P46=1

55. ¿Mejora con la actividad física/el ejercicio *(tiene el dolor pero después de que está un rato moviéndose, se le alivia)*? 1. Sí 0. No
ENTREV: si indica que "a veces" o "parece que sí", no insistir y marcar "sí".

PROG.: P.54 Y 55 EN LA MISMA PANTALLA

ENTREV.: En caso de obtener la misma respuesta a ambas cuestiones (sí a las dos o no, a las dos. Confirmad con el entrevistado/a la comprensión de la pregunta).

SÓLO SI P42=1 Y P46=1

56. ¿Mejora con el reposo? 1. Sí 0. No

ENTREV: si indica que "a veces" o "parece que sí", no insistir y marcar "sí".

SÓLO SI P42=1 Y P48=1

57. El dolor de rodilla, ¿empeora con el esfuerzo, al caminar o al subir/bajar escaleras *(si cuando levanta peso o cuando trabaja mucho, se encuentra peor)*?

1. Sí   0. No

ENTREV: si indica que "a veces" o "parece que sí", no insistir y marcar "sí".

SÓLO SI P42=1 Y P48=1

58. ¿Mejora con la actividad física/el ejercicio *(tiene el dolor pero después de que está un rato moviéndose, se le alivia)*?   1. Sí   0. No

ENTREV: si indica que "a veces" o "parece que sí", no insistir y marcar "sí".

PROG.: P.57 Y 58 EN LA MISMA PANTALLA

ENTREV.: En caso de obtener la misma respuesta a ambas cuestiones (sí a las dos o no, a las dos. Confirmad con el entrevistado/a la comprensión de la pregunta).

SÓLO SI P42=1 Y P48=1

59. ¿Mejora con el reposo *(se encuentra mejor cuando está sentado o acostado)*?

ENTREV: si indica que "a veces" o "parece que sí", no insistir y marcar "sí".

1. Sí   0. No

SÓLO SI P42=1 Y P50=1

60. El dolor de tobillo, ¿empeora con el esfuerzo, al caminar o al subir/bajar escaleras *(si cuando levanta peso o cuando trabaja mucho, se encuentra peor)*?   1. Sí   0. No

ENTREV: si indica que "a veces" o "parece que sí", no insistir y marcar "sí".

SÓLO SI P42=1 Y P50=1

61. ¿Mejora con la actividad física/el ejercicio (*tiene el dolor pero después de que está un rato moviéndose, se le alivia)*?   1. Sí   0. No

ENTREV: si indica que "a veces" o "parece que sí", no insistir y marcar "sí".

PROG.: P.60 Y 61 EN LA MISMA PANTALLA

ENTREV.: En caso de obtener la misma respuesta a ambas cuestiones (sí a las dos o no, a las dos. Confirmad con el entrevistado/a la comprensión de la pregunta)

SÓLO SI P42=1 Y P50=1

62. ¿Mejora con el reposo *(se encuentra mejor cuando está sentado o acostado)*?

ENTREV: si indica que "a veces" o "parece que sí", no insistir y marcar "sí".

1. Sí   0. No

SÓLO SI P42=1 Y P52=1

63. El dolor de pie, ¿empeora con el esfuerzo, al caminar o al subir/bajar escaleras *(si cuando levanta peso o cuando trabaja mucho, se encuentra peor)*?   1. Sí   0. No

ENTREV: si indica que "a veces" o "parece que sí", no insistir y marcar "sí".

SÓLO SI P42=1 Y P52=1

64. ¿Mejora con la actividad física/el ejercicio *(tiene el dolor pero después de que está un rato moviéndose, se le alivia)*?   1. Sí   0. No

ENTREV: si indica que "a veces" o "parece que sí", no insistir y marcar "sí".

PROG.: P.63 Y 64 EN LA MISMA PANTALLA
ENTREV.: En caso de obtener la misma respuesta a ambas cuestiones (sí a las dos o no, a las dos. Confirmad con el entrevistado/a la comprensión de la pregunta)

SÓLO SI P42=1 Y P52=1

65. ¿Mejora con el reposo *(se encuentra mejor cuando está sentado o acostado)*?
   1. Sí 0. No

   ENTREV: si indica que "a veces" o "parece que sí", no insistir y marcar "sí".

SÓLO SI P42=1 Y P44=1

66. El dolor de mano, ¿empeora al usar las manos/mover los dedos *(tiene más dolor cuando trabaja con las manos o hace esfuerzo con las manos)*? 1. Sí 0. No

   ENTREV: si indica que "a veces" o "parece que sí", no insistir y marcar "sí".

SÓLO SI P42=1 Y P44=1

67. ¿Mejora al usar las manos/mover los dedos *(el dolor mejora después de un rato moviendo las manos)*? 1. Sí 0. No

   ENTREV: si indica que "a veces" o "parece que sí", no insistir y marcar "sí".

PROG.: P.66 Y 67 EN LA MISMA PANTALLA
ENTREV.: En caso de obtener la misma respuesta a ambas cuestiones (sí a las dos o no, a las dos. Confirmad con el entrevistado/a la comprensión de la pregunta)

SÓLO SI P42=1 Y P44=1

68. ¿Mejora con el reposo *(mejora cuando tienes las manos quietas, sin hacer esfuerzos)*?
   1. Sí 0. No

   ENTREV: si indica que "a veces" o "parece que sí", no insistir y marcar "sí".

SÓLO SI P42=1

69. ¿Le ocurre que por la mañana no puede mover la zona afectada o la encuentra rígida? *(es decir, cuando se levanta por la mañana siente rigidez y le cuesta mover las articulaciones. Le cuesta caminar durante un rato si el dolor es en caderas, rodillas o pies, y si es en las manos, le cuesta cerrarlas al levantarse)* 1. Sí 0. No

SÓLO SI P42=1 Y P69=1

70. ¿Durante más de media hora? 1. Sí 0. No

ANTECEDENTES DE PSORIASIS

¿Le ha dicho su médico que tiene o ha tenido psoriasis *(lesiones en la piel rojas y escamosas que se localizan en los codos, rodillas, cuero cabelludo)*? 1. Sí 0. No

¿A algún familiar cercano (abuelos, padres, hermanos o hijos) le han diagnosticado psoriasis?
1. Sí 0. No 9. No sabe/no contesta

CUESTIONARIO TELEFÓNICO PARA LA VALORACIÓN DE LA SOSPECHA DE APs POR EL REUMATÓLOGO

Las preguntas variaban según si el cribado inicial había sido positivo para lumbalgia inflamatoria, artritis o entesitis. Si el cribado había sido positivo para más de una de ellas, mantener la sospecha para una era suficiente.

CRIBADO INCIAL POSITIVO PARA LUMBALGIA INFLAMATORIA

1. Fecha de la entrevista telefónica:
2. De acuerdo con el cuestionario realizado en (decir la fecha de la entrevista hecha por el call center), tiene o ha tenido dolor en la parte baja de la espalda/cintura/espalda a la altura de los riñones/en la zona lumbar/en las lumbares/lumbago o lumbalgia, sin que haya sido por un golpe / caída o sobresfuerzo. ¿Es así?
   ☐ Sí ☐ No

   Si respuesta afirmativa a la pregunta 2, continuar preguntando:
3. ¿Ha durado ese dolor 3 o más meses seguidos, aunque el dolor no sea fijo / tenga altibajos? ☐ Sí ☐ No

   Si respuesta afirmativa a la pregunta 3, continuar preguntando:
4. ¿Mejora con la actividad física y el ejercicio? ☐ Sí ☐ No
5. ¿Mejora con el reposo? ☐ Sí ☐ No
6. ¿Le despierta el dolor por la noche? ☐ Sí ☐ No
7. ¿Empezó antes de los 45 años? ☐ Sí ☐ No
8. ¿Le ha dicho su médico que tiene o ha tenido:
   - ☐ Psoriasis
   - ☐ Enfermedad inflamatoria intestinal (colitis ulcerosa/enfermedad de Crohn)
   - ☐ Uveítis (posible aclaración para el sujeto: ponerse el ojo rojo y doloroso que haya tenido que ir al oculista y que no fuese por infección)
   - ☐ Ninguna de las anteriores
9. A algún familiar cercano (abuelos, padres, hermanos o hijos) le han diagnosticado:
   - ☐ Psoriasis
   - ☐ Espondilitis anquilosante
   - ☐ Enfermedad inflamatoria intestinal/colitis ulcerosa/enfermedad de Crohn
   - ☐ Ninguna de las anteriores

   Teniendo en cuenta las respuestas anteriores:
10. Resultado del cribado de artritis psoriásica[1] ☐ Positivo ☐ Negativo
11. ¿Mantiene usted la sospecha de artritis psoriásica? ☐ Sí ☐ No
12. Motivo de la sospecha negativa (indicar en caso de que se decida no mantener la sospecha cuando el resultado del cribado es positivo de acuerdo a los criterios definidos por el comité científico):

    Si NO se mantiene la sospecha de artritis psoriásica:
13. ¿Existe sospecha de lumbalgia inflamatoria? ☐ Sí ☐ No

CRIBADO INCIAL POSITIVO PARA ARTRITIS

6. Fecha de la entrevista telefónica:

7. Localización del dolor por el cuestionario del Call Center
   Mano ☐ Sí ☐ No
   Cadera ☐ Sí ☐ No
   Rodilla ☐ Sí ☐ No
   Tobillo ☐ Sí ☐ No
   Pie ☐ Sí ☐ No
8. De acuerdo con el cuestionario realizado en (*decir la fecha de la entrevista hecha por el call center*), usted tiene o ha tenido dolor en (*decir la localización por el cuestionario del call center*), sin que haya recibido golpe/caída o sobreesfuerzo. ¿Es así?
   ☐ Sí ☐ No
   Si respuesta afirmativa a la pregunta 8, continuar preguntando:
9. ¿Le ha durado el dolor más de 4 semanas seguidas? ☐ Sí ☐ No
   Si respuesta afirmativa a la pregunta 9, continuar preguntando:
10. Localización:
    Mano ☐ Sí ☐ No
    Lateralidad ☐ Ambas ☐ Derecha ☐ Izquierda
    Cadera ☐ Sí ☐ No
    Lateralidad ☐ Ambas ☐ Derecha ☐ Izquierda
    Rodilla ☐ Sí ☐ No
    Lateralidad ☐ Ambas ☐ Derecha ☐ Izquierda
    Tobillo ☐ Sí ☐ No
    Lateralidad ☐ Ambas ☐ Derecha ☐ Izquierda
    Pie ☐ Sí ☐ No
    Lateralidad ☐ Ambas ☐ Derecha ☐ Izquierda

11. ¿Ha tendido inflamación/hinchazón de forma mantenida en (*decir la localización afectada*) más de 4 semanas? ☐ Sí ☐ No

12. El dolor de cadera/ingle, ¿empeora con el esfuerzo, al caminar o subir/bajar escaleras?
    ☐ Sí ☐ No
    ó
13. ¿mejora con la actividad física/el ejercicio? ☐ Sí ☐ No
14. ¿Mejora el dolor de cadera/ingle con el reposo? ☐ Sí ☐ No

15. El dolor de rodilla(s), ¿empeora con el esfuerzo, al caminar o subir/bajar escaleras?
    ☐ Sí ☐ No
    ó
16. ¿mejora con la actividad física/el ejercicio? ☐ Sí ☐ No
17. ¿Mejora el dolor de rodilla(s) con el reposo? ☐ Sí ☐ No

18. El dolor de tobillo, ¿empeora con el esfuerzo, al caminar o subir/bajar escaleras?
    ☐ Sí ☐ No
    ó
19. ¿mejora con la actividad física/el ejercicio? ☐ Sí ☐ No
20. ¿Mejora el dolor de tobillo con el reposo? ☐ Sí ☐ No

21. ¿El dolor de pie ¿empeora con el esfuerzo, al caminar o subir/bajar escaleras?
    ☐ Sí ☐ No
    ó
22. ¿mejora con la actividad física/el ejercicio? ☐ Sí ☐ No
23. ¿Mejora el dolor de pie con el reposo? ☐ Sí ☐ No

24. El dolor de mano(s), ¿empeora al usar las manos/mover los dedos? ☐ Sí ☐ No

ó

25. ¿mejora al usar las manos/mover los dedos? ☐ Sí ☐ No
26. ¿Mejora el dolor de mano(s) con el reposo? ☐ Sí ☐ No
27. ¿Le ocurre que por la mañana no puede mover la zona(s) afectada(s) o la(s) encuentra rígida(s)? ☐ Sí ☐ No

    Si respuesta afirmativa a la pregunta 27, preguntar:
28. ¿Durante más de media hora? ☐ Sí ☐ No

    Teniendo en cuenta las respuestas anteriores:
29. Resultado cribado artritis[1] ☐ Positivo ☐ Negativo
30. ¿Mantiene usted la sospecha de artritis? ☐ Sí ☐ No
31. Motivo de la sospecha negativa de artritis (indicar en caso de que se decida no mantener la sospecha cuando el resultado del cribado es positivo de acuerdo a los criterios definidos por el comité científico):

    Si hay sospecha de artritis, continuar preguntando:
32. ¿Le ha dicho su médico que tiene o ha tenido psoriasis? ☐ Sí ☐ No
33. ¿A algún familiar cercano (abuelos, padres, hermanos o hijos) le han diagnosticado psoriasis? ☐ Sí ☐ No

    Teniendo en cuenta las respuestas anteriores:
34. ¿Mantiene usted la sospecha de artritis psoriásica? ☐ Sí ☐ No

CRIBADO INCIAL POSITIVO PARA ENTESITIS

1. Fecha de la entrevista telefónica (dd/mm/aaaa): __ __/__ __/__ __ __ __
2. ¿Le duele o le ha dolido el talón, o lo ha notado hinchado (sin que haya sido por un golpe o sobresfuerzo)?

   ☐ Sí ☐ No

   Si respuesta afirmativa a la pregunta 2, continuar preguntando:
3. ¿Le ha dicho su médico que tiene o ha tenido psoriasis? ☐ Sí ☐ No
4. ¿A algún familiar cercano (abuelos, padres, hermanos o hijos) le han diagnosticado psoriasis? ☐ Sí ☐ No

   Teniendo en cuenta las respuestas anteriores:
5. ¿Se mantiene la sospecha de artritis psoriásica? ☐ Sí ☐ No

[1] De acuerdo con los criterios definidos por el comité científico: los criterios utilizados por el reumatólogo para mantener la sospecha deben ser los mismos que los utilizados en el cribado inicial, ya que se consideraron suficientemente específicos.
