# Supplementary material for: Higher prevalence of psoriatic arthritis in the adult population in Spain? A population-based cross-sectional study
Source: PLoS One. 2020 Jun 17;15(6):e0234556. doi: 10.1371/journal.pone.0234556 (PMC7299392; doi:10.1371/journal.pone.0234556)
Supplement: S3 File — (PDF) [file pone.0234556.s003.pdf]

```
* Encoding: UTF-8.
*Weighting.

if (group=1 & sex2 = 1 & agegroup = 1 ) WEIGHT_GroupCCAA = 2922.59016393443.
if (group=1 & sex2 = 1 & agegroup = 2 ) WEIGHT_GroupCCAA = 2940.56544502618.
if (group=1 & sex2 = 1 & agegroup = 3 ) WEIGHT_GroupCCAA = 4300.27857142857.
if (group=1 & sex2 = 1 & agegroup = 4 ) WEIGHT_GroupCCAA = 7583.45070422535.
if (group=1 & sex2 = 1 & agegroup = 5 ) WEIGHT_GroupCCAA = 6908.88888888889.
if (group=1 & sex2 = 1 & agegroup = 6 ) WEIGHT_GroupCCAA = 8400.02857142857.
if (group=1 & sex2 = 1 & agegroup = 7 ) WEIGHT_GroupCCAA = 5777.8.
if (group=1 & sex2 = 2 & agegroup = 1 ) WEIGHT_GroupCCAA = 6193.28571428571.
if (group=1 & sex2 = 2 & agegroup = 2 ) WEIGHT_GroupCCAA = 5544.
if (group=1 & sex2 = 2 & agegroup = 3 ) WEIGHT_GroupCCAA = 3833.47741935484.
if (group=1 & sex2 = 2 & agegroup = 4 ) WEIGHT_GroupCCAA = 3277.91764705882.
if (group=1 & sex2 = 2 & agegroup = 5 ) WEIGHT_GroupCCAA = 3929.85833333333.
if (group=1 & sex2 = 2 & agegroup = 6 ) WEIGHT_GroupCCAA = 3769.9175257732.
if (group=1 & sex2 = 2 & agegroup = 7 ) WEIGHT_GroupCCAA = 6337.29310344828.
if (group=2 & sex2 = 1 & agegroup = 1 ) WEIGHT_GroupCCAA = 7779.81818181818.
if (group=2 & sex2 = 1 & agegroup = 2 ) WEIGHT_GroupCCAA = 8533.57142857143.
if (group=2 & sex2 = 1 & agegroup = 3 ) WEIGHT_GroupCCAA = 10529.5049019608.
if (group=2 & sex2 = 1 & agegroup = 4 ) WEIGHT_GroupCCAA = 18437.6630434783.
if (group=2 & sex2 = 1 & agegroup = 5 ) WEIGHT_GroupCCAA = 14344.6395348837.
if (group=2 & sex2 = 1 & agegroup = 6 ) WEIGHT_GroupCCAA = 11664.1857142857.
if (group=2 & sex2 = 1 & agegroup = 7 ) WEIGHT_GroupCCAA = 11417.9512195122.
if (group=2 & sex2 = 2 & agegroup = 1 ) WEIGHT_GroupCCAA = 15988.4269662921.
if (group=2 & sex2 = 2 & agegroup = 2 ) WEIGHT_GroupCCAA = 12729.2151898734.
if (group=2 & sex2 = 2 & agegroup = 3 ) WEIGHT_GroupCCAA = 8062.078125.
if (group=2 & sex2 = 2 & agegroup = 4 ) WEIGHT_GroupCCAA = 6596.75954198473.
if (group=2 & sex2 = 2 & agegroup = 5 ) WEIGHT_GroupCCAA = 7388.28729281768.
if (group=2 & sex2 = 2 & agegroup = 6 ) WEIGHT_GroupCCAA = 7766.0859375.
if (group=2 & sex2 = 2 & agegroup = 7 ) WEIGHT_GroupCCAA = 12701.125.
if (group=3 & sex2 = 1 & agegroup = 1 ) WEIGHT_GroupCCAA = 6154.40677966102.
if (group=3 & sex2 = 1 & agegroup = 2 ) WEIGHT_GroupCCAA = 6069.1387283237.
if (group=3 & sex2 = 1 & agegroup = 3 ) WEIGHT_GroupCCAA = 10621.5533980583.
if (group=3 & sex2 = 1 & agegroup = 4 ) WEIGHT_GroupCCAA = 13637.7611940299.
```

```
if (group=3 & sex2 = 1 & agegroup = 5 ) WEIGHT_GroupCCAA = 6020.35779816514.
if (group=3 & sex2 = 1 & agegroup = 6 ) WEIGHT_GroupCCAA = 10295.4545454545.
if (group=3 & sex2 = 1 & agegroup = 7 ) WEIGHT_GroupCCAA = 7647.2380952381.
if (group=3 & sex2 = 2 & agegroup = 1 ) WEIGHT_GroupCCAA = 14097.431372549.
if (group=3 & sex2 = 2 & agegroup = 2 ) WEIGHT_GroupCCAA = 11307.4505494506.
if (group=3 & sex2 = 2 & agegroup = 3 ) WEIGHT_GroupCCAA = 5496.1269035533.
if (group=3 & sex2 = 2 & agegroup = 4 ) WEIGHT_GroupCCAA = 5379.06896551724.
if (group=3 & sex2 = 2 & agegroup = 5 ) WEIGHT_GroupCCAA = 5762.64516129032.
if (group=3 & sex2 = 2 & agegroup = 6 ) WEIGHT_GroupCCAA = 6385.09090909091.
if (group=3 & sex2 = 2 & agegroup = 7 ) WEIGHT_GroupCCAA = 9159.5.


*PsA prevalence.

COMPUTE Prev_PsA=0.
IF ((dxPsA1=1) OR (dxPsA2='TRUE')) Prev_PsA=1.
IF (state_PsA = 'Open' & missing = 1) Prev_PsA=$SYSMIS.
VALUE LABELS Prev_PsA 0 'No' 1 'Yes'.
```
